# Synergistic effect of angiotensin II on vascular endothelial growth factor-A-mediated differentiation of bone marrow-derived mesenchymal stem cells into endothelial cells

**DOI:** 10.1186/scrt538

**Published:** 2015-01-06

**Authors:** Izuagie Attairu Ikhapoh, Christopher J Pelham, Devendra K Agrawal

**Affiliations:** Department of Medical Microbiology and Immunology, Creighton School of Medicine, 2500 California Plaza, Omaha, NE 68178-0405 USA; Department of Biomedical Sciences, Creighton School of Medicine, 2500 California Plaza, Omaha, NE 68178 USA; Center for Clinical and Translational Science, CRISS II Room 510, Creighton School of Medicine, 2500 California Plaza, Omaha, NE 68178-0405 USA

## Abstract

**Introduction:**

Increased levels of angiotensin II (Ang II) and activity of Ang II receptor type 1 (AT1R) elicit detrimental effects in cardiovascular disease. However, the role of Ang II receptor type 2 (AT2R) remains poorly defined. Mesenchymal stem cells (MSCs) replenish and repair endothelial cells in the cardiovascular system. Herein, we investigated a novel role of angiotensin signaling in enhancing vascular endothelial growth factor (VEGF)-A-mediated differentiation of MSCs into endothelial cells (ECs).

**Methods:**

Bone marrow was aspirated from the femurs of Yucatan microswine. MSCs were extracted via ficoll density centrifugation technique and were strongly immunopositive for MSC markers, CD44, CD90, and CD105, but negative for hematopoietic markers, CD14 and CD45. Subsequently, naïve MSCs were differentiated for 10 days in varying concentrations and combinations of VEGF-A, Ang II, and AT1R or AT2R antagonists. Markers specific to ECs were determined by FACS analysis.

**Results:**

AT1R and AT2R expression and cellular localization was demonstrated in MSCs stimulated with VEGF-A and Ang II via quantitative RT-PCR and immunofluorescence, respectively. Differentiation of naïve MSCs in media containing Ang II (2 ng/ml) plus low-dose VEGF-A (2 ng/ml) produced a significantly higher percentage of cells that were positive for expression of EC markers (for example, platelet endothelial cell adhesion molecule, vascular endothelial Cadherin and von Willebrand factor) compared to VEGF-A alone. Ang II alone failed to induce EC marker expression. MSCs differentiated with the combination of Ang II and VEGF-A were capable of forming capillary tubes using an *in vitro* angiogenesis assay. Induction of EC marker expression was greatly attenuated by co-treatment of Ang II/VEGF-A with the AT2R antagonist PD123319, but not the AT1R antagonist telmisartan.

**Conclusions:**

We report the presence of functional AT2R receptor on porcine bone marrow-derived MSCs, where it positively regulates EC differentiation. These findings have significant implications toward therapeutic approaches based on activation of AT2R, which could be a means to stimulate regeneration of damaged endothelium and prevent vascular thrombosis.

## Introduction

Occlusive cardiovascular diseases are the foremost cause of mortality in the United States, totaling more than 33% of deaths per year with 2,200 fatalities per day [1, 2]. Development of atherosclerotic plaque and intimal thickening in carotid and coronary arteries are dominant predictors of future myocardial infarction [3]. Following myocardial infarction and/or ischemia, interventional procedures, including angioplasty and stenting, are performed. Endothelial dysfunction remains an inherent secondary effect of these procedures [4]. Deployment of drug-eluting stents in coronary arteries causes endothelial cell wasting, which contributes to neointimal hyperplasia of the underlying smooth muscle cells, restenosis of the artery and even in-stent thrombosis. Following angioplasty and stent replacement, reocclusion rates are as high as 20% of total procedures performed per year [5]. The high incidence of complications due to restenosis is a large burden on healthcare cost. Even worse, acute coronary thrombosis is a cause of sudden fatalities [6].

Cell-based therapies have been explored as treatments for heart disease [7]. In particular, mesenchymal stem cell (MSC)-based treatments have been proposed as a potential method for regenerating and/or rejuvenating dysfunctional endothelium [8]. MSCs are multipotent cells capable of differentiating into cells of mesodermal lineage [9]. Vascular endothelial growth factor (VEGF-A) is the best-defined growth factor that promotes differentiation of MSCs into endothelial cells (ECs) [10]. VEGF-A is an EC mitogen that plays an essential role in both vasculogenesis and angiogenesis. VEGF-A interaction with its cognate tyrosine kinases induces multiple pro-angiogenic pathways that promote cell survival, migration, and proliferation [11, 12]. Indeed, stimulation of VEGF receptor 2 on bone marrow-derived mesenchymal stem cells (BM-MSCs) by treatment with recombinant VEGF-A is an efficient way to induce differentiation of cultured MSCs into ECs *in vitro*[13].

To utilize MSCs in endothelial regeneration *in vivo*, it is critical to identify molecular agonists that can regulate the differentiation of MSCs into EC-like cells [14, 15]. The angiotensin II (Ang II) receptors AT1R and AT2R are G protein-coupled receptors (GPCRs) of great importance to cardiovascular function. The precursor angiotensinogen is cleaved by renin to produce angiotensin I, which is further cleaved to Ang II by angiotensin-converting enzyme [16]. Ang II binds and activates AT1R and AT2R, which are both expressed in important cardiovascular tissues, including vascular smooth muscle cells and ECs [17].

Through AT1R and AT2R, Ang II has divergent effects that are critical for vascular homeostasis. Ang II stimulates vascular smooth muscle contraction of the coronary blood vessels [18]. At the same time, Ang II can induce endothelium-dependent production of the vasodilator nitric oxide [19–21]. AT1R and AT2R could therefore play an important role in the differentiation of MSCs into ECs. In this study, we show expression of ATR1 and ATR2 on porcine bone BM-MSCs. We demonstrate that activation of AT2R elicits a synergistic response with VEGF-A to augment the differentiation of BM-MSCs to ECs. These findings have significant clinical implications with regard to the effects of medications that inhibit Ang II production or activation of AT2R on endothelial function or regeneration. Optimization of protocols for EC differentiation will be vital for cell-based therapies aimed at repairing damaged endothelium.

## Materials and methods

### Mesenchymal stem cell isolation and differentiation

MSCs were isolated, characterized, and differentiated from Yucatan microswine femurs as reported previously by our group [22]. All animal procedures were in compliance with applicable federal, state, and local laws and regulations, and institutional policies. The Institutional Animal Care and Use Committee of Creighton University approved the animal research protocol. Cells used for experiments in this study were between passages 3 and 5. The isolated MSCs were CD14^–^CD45^–^CD44^+^CD90^+^CD105^+^. The growth media used to harvest and culture MSCs was Dulbecco’s modified Eagle’s medium with 10% fetal bovine serum. The differentiation media (DM) used for differentiation was endothelial growth media 2 (EGM-2) containing 2 ng/ml, 25 ng/ml, or 50 ng/ml recombinant human VEGF-A_165_ (Peprotech, Rocky Hill, NJ, USA), and/or 2 ng/ml, 25 ng/ml, or 50 ng/ml recombinant human Ang II (Sigma; St. Louis, MO, USA). Basic EGM-2 was used as negative control DM. Stimulation began when MSCs were at 50% confluency and continued for 10 days. The cell cultures were maintained at 37°C in a humidified atmosphere containing 5% carbon dioxide. Media containing varying concentrations of VEGF-A, Ang II, and ATR inhibitor were changed every 48 hours.

### Fluorescence-activated cell sorting characterization of naïve MSCs and ECs

Briefly, cells (1 × 10^6^/ml) were washed with phosphate-buffered saline (PBS) containing 4% fetal bovine serum and incubated with primary antibodies conjugated to fluorophore (fluorescein isothiocyanate for 30 minutes at 4°C in the dark. The antibody concentrations followed the specifications of the manufacturer. The cells were further washed three times in PBS and resuspended in 750 μl FACS-FIX. Flow cytometry was performed on a BD FACSAria I/II System (BD Biosciences, San Jose, CA, USA). Naïve MSCs highly expressed stem cell markers CD44, CD90 and CD105. The cells from the same gate were negative for the macrophage marker CD14 and the hematopoietic stem cell marker CD45. After differentiation, MSCs were analyzed for the EC markers platelet endothelial cell adhesion molecule-1 (PECAM)–fluorescein isothiocyanate (ebiosciences, San Diego, CA, USA), vascular endothelial cadherin (VE-cadherin)–fluorescein isothiocyanate (ebiosciences), and von Willebrand factor (vWF)–fluorescein isothiocyanate (ebiosciences).

### Transfection

For small interfering RNA (siRNA)-mediated knockdown experiments, MSCs were transfected using the Amaxa Nucleofector II Device with the MSC Nucleofector Kit (Lonza, Basel, Switzerland) according to the manufacturer’s optimized protocol for MSCs. For each nucleofection sample, we harvested 2.5 × 10^5^ cells (cell counter; Beckman Coulter, Brea, CA; USA). Briefly, MSCs were washed in Dulbecco’s modified Eagle’s medium supplemented with 200 U/ml penicillin G sulfate plus 200 mg/ml streptomycin sulfate. Nucleofector medium (100 μl) containing siRNA (5 to 50 nM; Origene, Rockville, MD, USA) was administered in a single square wave pulse in a 4 mm diameter cuvette. After electroporation, MSCs were washed with PBS three times, then transferred into DM or growth media and incubated at 37°C. For transfection control, an equivalent amount of scrambled siRNA was used in either DM. At 72 hours post transfection, cell cultures were harvested and protein lysates were isolated for western blot analysis.

### Western blot

Protein fractions were isolated using the active motif kit (Active Motif, Carlsbad, CA, USA). Total protein lysates were quantified by Bradford assay. Proteins were separated by 10% SDS-PAGE, transferred onto a nitrocellulose membrane, and blocked overnight in blocking solution (1× TBS, pH 7.6, 0.1% Tween-20, and 5% w/v nonfat dry milk). The membrane was then incubated with antibodies specific for VEGF-A (Ab105846, 1:500; Abcam; Cambridge, MA, USA). As a loading control, the membrane was probed for glyceraldehyde-3-phosphate dehydrogenase (GAPDH) (1:1,000, NB300-221; NOVUS Biological; Littleton, CO, USA). The membrane was then incubated with horseradish peroxidase-conjugated secondary antibody (1:1,000) in blocking solution for 1 hour at room temperature. Horseradish peroxidase activity was detected by incubating the membrane in chemiluminescence solution (Bio-Rad, Hercules, CA, USA). The exposure time was adjusted to keep the integrated optical densities within a linear and nonsaturated range. Densitometric analysis was carried out using a UVP Bioimaging system (UVP, Minneapolis, MN, USA).

### Quantitative RT-PCR

Total RNA was isolated from naïve MSCs using Trizol reagent (Sigma) according to the manufacturer’s instructions. The yield of RNA was quantified using a Nanodrop (Thermo-Scientific, Rockford, IL, USA). First-strand cDNA synthesis was performed following the manufacturer’s instructions (Improm II reverse transcription kit; Promega, Madison, WI, USA) using oligo dT primers. Real-time quantitative PCR was performed using SYBR Green Master Mix and a Real-time PCR system (CFX96; BioRad Laboratories, Hercules, CA, USA). The following primers are reportedly specific to swine [23]: AT1R-F, 5′-GGCCAGTGTTTTTCTTTTGAATTTAGCAC-3′ and AT1R-R, 5′-TGAACAATAGCCAGGTATCGATCAATGC-3′; AT2R-F, 5′-GTTCCCCTTGTTTGGTGTAT-3′ and AT2R-R, 5′-CATCTTCAGGACTTGGTCAC-3′; and GAPDH-F, 5′-CCCATCACCATCTTCCAGGAG-3′ and GAPDH-R, 5′-GTTGTCATGGATGACCTTGGCC-3′.

Primers were obtained from Integrated DNA Technologies (Coralville, IA, USA). The specificity of the primers was confirmed by running a melting curve (data not shown). The thermocycler conditions were as follows: 5 minutes at 95°C for initial denaturation, and 40 cycles of 30 seconds at 95°C, 30 seconds at 55 to 60°C (depending upon the primer annealing temperatures), and 30 seconds at 72°C. Fold expression of mRNA transcripts relative to controls was determined after normalizing to GAPDH.

### Angiotensin receptor blockade

To analyze the effects of AT1R and AT2R on differentiation, MSCs were pre-incubated with 5 μM Telmisartan (an AT1R blocker) or 5 μM PD123319 (an AT2R blocker) for 1 hour. Pretreated MSCs were then cultured in DM containing 1 μM of either ATR blocker. In preliminary experiments, titration was performed for AT1R-specific and AT2R-specific antagonists (1 to 20 μM) for 24 hours to determine the optimal concentration that had no effect on cell viability or proliferation. Cells were then detached by with 0.25% trypsin/ethylenediamine tetraacetic acid, counted, and evaluated by annexin V/PI assay. Florescence-activated cell sorting analysis revealed that >95% of cell were viable at concentrations of ATR blockers between 1 μM and 5 μM. MSCs were then induced to undergo differentiation with differentiating media containing the indicated concentrations of VEGF-A alone or in combination with Ang II and/or ATR antagonists.

### Immunofluorescence

Cells were incubated in blocking solution containing PBS, 0.25% Triton X-100, 10 mg/ml bovine serum albumin, and 5% normal goat serum (Jackson Laboratories, West Grove, PA, USA) for 1 hour at room temperature. The cells were incubated with primary antibodies selective for anti-AT1R (Ab9391, 1:1,000; abcam) or anti-AT2R (Ab19134, 1:1,000; abcam) for 1 hour at room temperature. After washing with PBS containing 0.1% bovine serum albumin three times for 5 minutes each, a secondary antibody (affinity purified goat anti-rabbit Cy2 and Cy3 antibody, 1:500, Jackson laboratories; West Grove, USA) was applied to the sections for 1 hour in the dark to visualize AT1R-labeled and AT2R-labeled cells (Jackson Immunolabs, West Grove, PA, USA). Negative controls were run in parallel either using rabbit pre-immune serum PAC-767 (Pacific Immunology, Ramona, CA, USA) instead of primary antibody or by complete omission of primary antibody. Negative control was absent of staining. Sections were washed with PBS with 0.1% bovine serum albumin three times for 5 minutes and dipped into distilled water for 2 seconds. Fluorescence was preserved by sealing specimens with a solution of equal parts of PBS and glycerol containing 10 mg/ml *n*-propyl gallate, and 1.5 mg/ml 4′,6-diamidino-2-phenylindole. To prevent the escape of the mounting medium from the cover slips, a single layer of nail polish was placed around the edges. Pictures were taken within 1 hour of mounting using an Olympus DP71 camera (Olympus, St Louis, MO, USA).

### Angiogenesis assay

After stimulation for 10 days, MSCs were harvested and an angiogenesis assay was performed according to the manufacturer’s protocol (Chemicon, Temecula, CA, USA). Polymerized EC matrices were prepared by incubating 100 μl ECMatrix gel solution into each well of a 24-well plate at 37°C for 1 hour. The stimulated cells were seeded at a concentration of 1 × 10^4^ cells on EC matrices. EGM-2 medium (300 μl) was added to each well and maintained at 37°C and 5% carbon dioxide incubation for 6 hours. The formation of capillary tubes was analyzed using an inverted phase contrast microscope (CKK41; Olympus).

### Statistical analysis

Data are presented as the mean ± standard deviation from three to six independent experiments. For each experiment, MSC cultures were isolated from the femoral bone of separate pigs. Data were analyzed using GraphPad Prism, GraphPad Software; La Jolla, California, USA. Multiple group comparisons were performed by Bonferroni’s multiple comparison tests using one-way analysis of variance. *P* <0.05 was accepted as statistically significant.

## Results

### Characterization of bone marrow-derived MSCs

**Figure 1:**
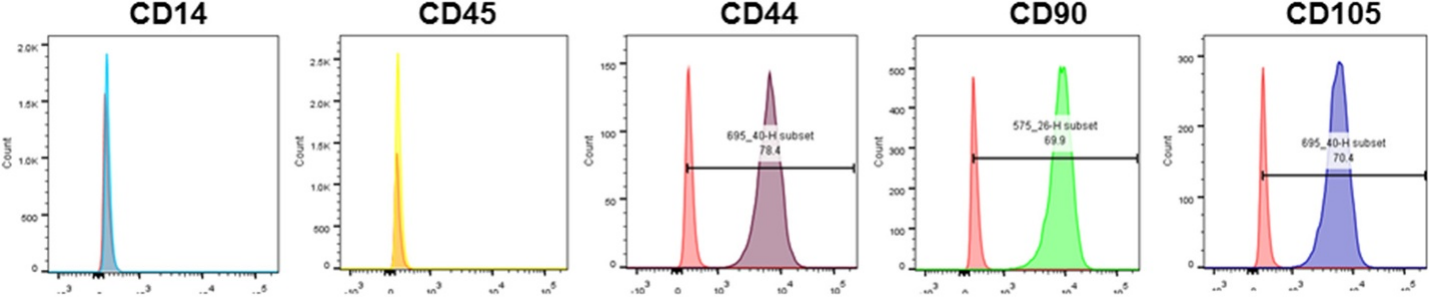
**Characterization of bone marrow-derived mesenchymal stem cells.** Flow cytometry data revealed that mesenchymal stem cells (MSCs) at passages 3 to 5 stained negatively for CD14 (monocyte marker) and CD45 (hematopoietic marker), but expressed surface markers that are indicative of MSC lineage, including CD44 (hyaluronic acid receptor), CD90 (Thy-1), and CD105 (Endoglin). Isolated MSCs exhibited stem-like properties.

Primary cultures of MSCs isolated from porcine bone marrow exhibited fibroblastoid morphology typical of MSCs [24]. Flow cytometry data revealed that cells at passages 3 to 5 stained negatively for CD14 (monocyte marker) and CD45 (hematopoietic marker) (Figure 1). The same MSCs expressed CD44 (hyaluronic acid receptor), CD90 (Thy-1), and CD105 (Endoglin), characteristic of MSCs (Figure 1).

### Expression of AT1R and AT2R on naïve MSCs

**Figure 2:**
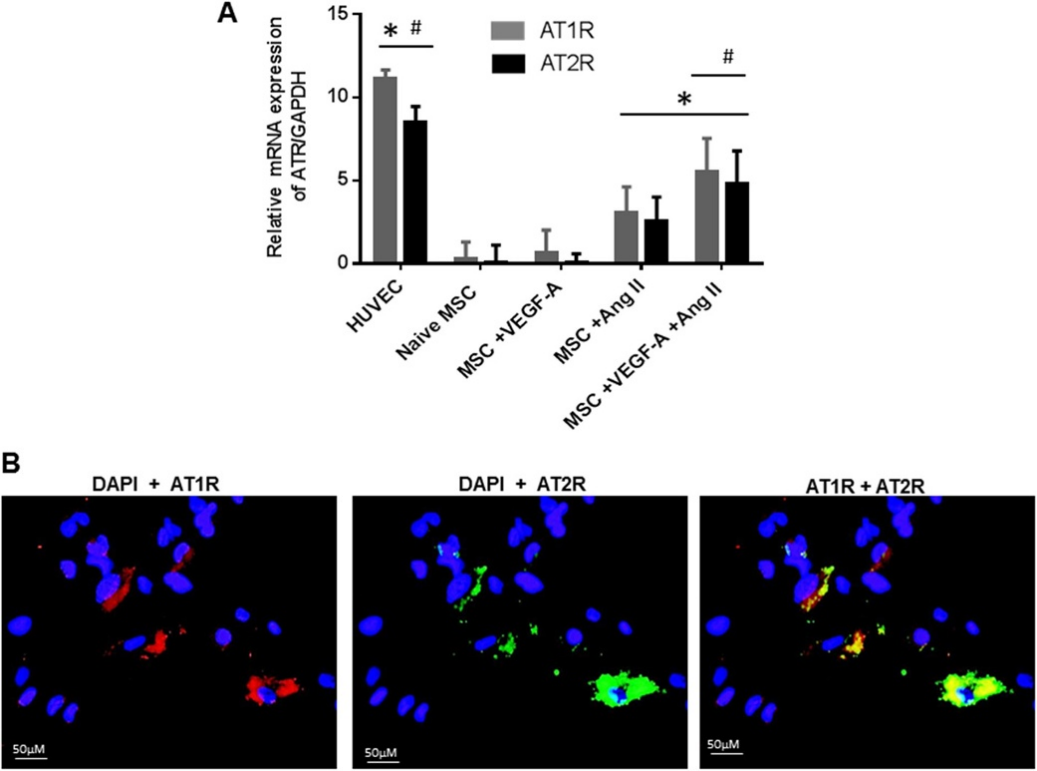
**Effect of VEGF-A and angiotensin II treatment on the expression of angiotensin receptors.** Bone marrow-derived mesenchymal stem cells (BM-MSCs) were cultured in vascular endothelial growth factor (VEGF-A; 2 ng/ml), angiotensin II (Ang II; 2 ng/ml), or combined VEGF-A/Ang II for 24 hours. Expression of angiotensin receptor AT1R and AT2R mRNA was analyzed via quantitative RT-PCR, and human umbilical vein endothelial cells (HUVECs) were used as positive control **(A)**. Immunostaining demonstrating co-localization of AT1R and AT2R on the cell membrane of BM-MSCs treated with VEGF-A (2 ng/ml) plus Ang II (2 ng/ml). AT1R stained in red co-localized with AT2R labeled in green to produce a yellow merge **(B)**. One image representative of three independent experiments is shown. Data are representative of three independent experiments performed from three different cultures from swine bone marrow. *P <0.05 vs. naïve MSCs. ^#^ *P* <0.05 vs. Ang II-treated MSCs, *n* = 3. DAPI, 4′,6-diamidino-2-phenylindole.

Control porcine BM-MSCs were cultured in basic EGM-2 control media containing 10% fetal bovine serum. Additional MSC cultures were stimulated with VEGF-A (2 ng/ml) alone, Ang II (2 ng/ml) alone, or the combination of VEGF-A/Ang II for 24 hours. Quantitative RT-PCR was used to analyze the mRNA expression of AT1R and AT2R on treated MSCs. The data reveal that the combination of VEGF-A and Ang II produced a greater induction in the mRNA expression of both AT1R and AT2R on differentiating MSCs than VEGF-A or Ang II alone (Figure 2A). Human umbilical vein endothelial cells (HUVECs) were used as an independent positive control for ECs. HUVEC AT1R and AT2R mRNA expression was significantly greater than that of treated or naïve MSCs (Figure 2A). Likewise, AT1R and AT2R expression on MSCs was confirmed by immunofluorescence using antibodies that have been validated for specificity [25, 26] (Figure 2B). AT1Rs and AT2Rs were expressed heterogeneously in the BM-MSC population and, in many cases, were co-expressed on the same cell (Figure 2B).

### VEGF-A induces dose-dependent expression of endothelial cell markers on MSCs

**Figure 3:**
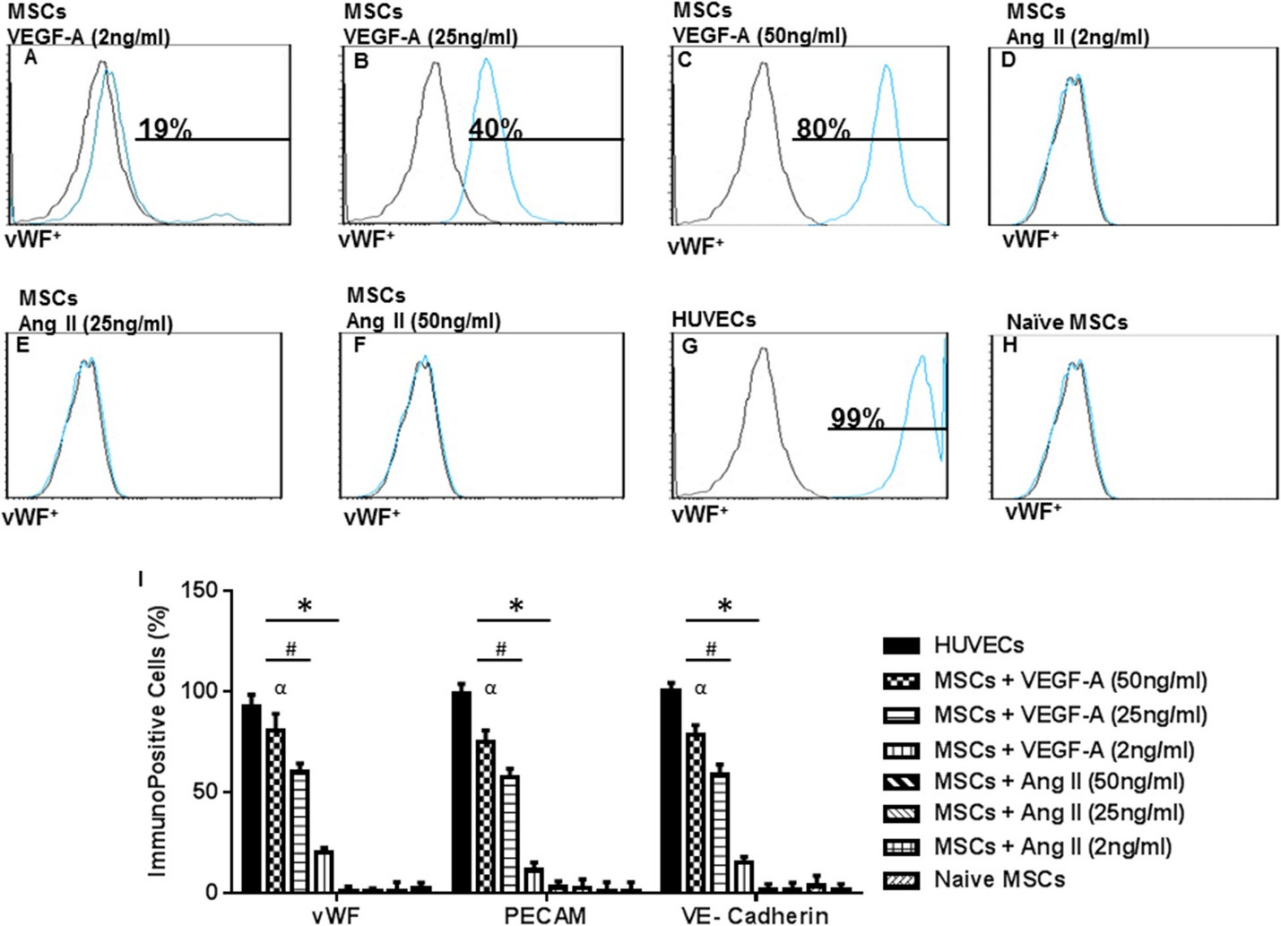
**Immunopositivity of endothelial cells differentiated from mesenchymal stem cells in the presence of VEGF-A for PECAM, vWF, and VE-cadherin.** Representative grids for von Willebrand factor (vWF) are shown. Low-dose vascular endothelial growth factor (VEGF-A) induced a small increase in immunopositivity for platelet endothelial cell adhesion molecule-1 (PECAM-1), vWF, and vascular endothelial cadherin (VE-cadherin) **(A, I)**. Higher concentrations of VEGF-A (25 to 50 ng/ml) induced a marked increase in expression of endothelial cell (EC) markers **(B, C, I)**. Angiotensin II (Ang II; 2 to 50 ng/ml) had no effect on the expression of EC markers **(D, E, F, I)**. Human umbilical vein endothelial cells (HUVECs) were used as an independent positive control showing ≈ 99% immunopositivity to vWF **(G, I)**. Naïve MSCs were negative for EC marker expression **(H, I)**. Graphical representation of the fluorescence-activated cell sorting (FACS) data **(I)**. Note that HUVECs were excluded from the statistical analyses. *P <0.05 vs. naïve MSCs. ^#^ *P* <0.05 vs. VEGF-A (2 ng/ml). ^α^ *P* <0.05 VEGF-A (50 ng/ml) vs. VEGF-A (25 ng/ml)-treated MSCs, *n* = 3. MSC, mesenchymal stem cell.

Our group previously reported that 50 ng/ml VEGF-A (high-dose VEGF-A) induces the expression of EC markers PECAM, vWF, and VE-cadherin [22]. Here, we demonstrate that dose-dependent treatment of BM-MSCs with 2 ng/ml, 25 ng/ml, and 50 ng/ml VEGF-A induces a coordinated increase in immunopositive expression of the EC markers PECAM-1, VE-cadherin, and vWF. Specifically, low-dose VEGF-A (2 ng/ml) resulted in a small increase in the percentage for vWF^+^ cells (16 ± 2%), PECAM-1^+^ cells (13 ± 4%) and VE-cadherin^+^ cells (11 ± 7%) (Figure 3A,I). An intermediate dose of VEGF-A (25 ng/ml) produced a higher positive percentage of vWF^+^ cells (39 ± 2%), PECAM-1^+^ cells (33 ± 2%), and VE-cadherin^+^ cells (43 ± 5) (Figure 3B,I). Finally, high-dose VEGF-A (50 ng/ml) produced the highest percentage of vWF^+^ cells (78 ± 1%), PECAM-1^+^ cells (61 ± 1%), and VE-cadherin^+^ cells (65 ± 1%) (Figure 3C,I). Differentiation media supplemented only with Ang II (2 to 50 ng/ml) had no significant effect on the expression of vWF, PECAM-1, and VE-cadherin (Figures 3D,E,F,I). HUVECs were used as a positive control for EC marker expression. HUVECs were highly immunopositive for PECAM-1, VE-cadherin, and vWF (>90% immunopositivity) (Figure 3G,I), whereas naïve BM-MSCs were negative for EC markers (Figure 3H,I).

### Synergistic effect of VEGF-A and angiotensin II

**Figure 4:**
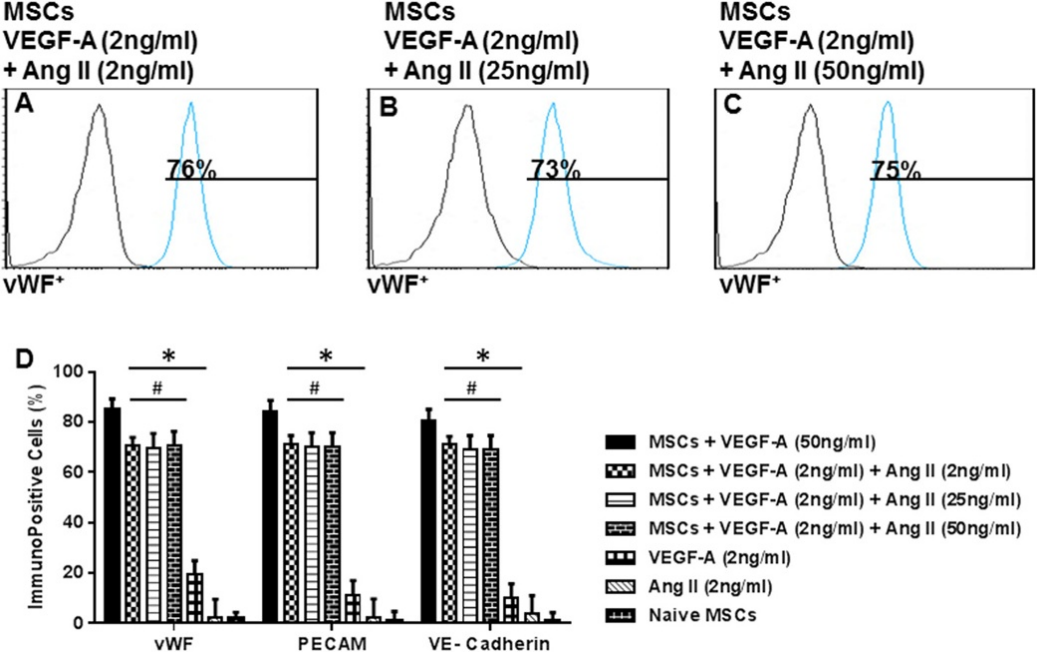
**Synergistic effect of angiotensin II and low-dose VEGF-A to promote mesenchymal stem cell differentiation into endothelial cells.** Co-culture of naïve bone marrow-derived mesenchymal stem cells (BM-MSC) with vascular endothelial growth factor (VEGF-A; 2 ng/ml) and angiotensin II (Ang II; 2 ng/ml) resulted in significantly increased expression of platelet endothelial cell adhesion molecule-1 (PECAM-1), von Willebrand factor (vWF), and vascular endothelial cadherin (VE-cadherin) **(A)**. Increasing the dose of Ang II in combination with the low dose of VEGF-A did not result in further increase in EC marker expression **(B, C, D)**. Note that human umbilical vein endothelial cells were excluded from the statistical analyses. *P <0.05 vs. naïve MSCs. ^#^ *P* <0.05 vs. VEGF-A (2 ng/ml)-treated MSCs, *n* = 3.

Low-dose VEGF-A (2 ng/ml) alone or Ang II alone (2, 25, or 50 ng/ml) failed to induce appreciable expression of EC markers (Figure 3I). However, co-treatment of naïve BM-MSCs with low-dose VEGF-A (2 ng/ml) in combination with Ang II caused a marked increase in the percentage of immunopositive cell expression for the EC markers tested (Figure 4A,B,C,D). Specifically, low-dose VEGF-A (2 ng/ml) and Ang II (2 ng/ml) enhanced immunopositive cell levels to 65 ± 2% vWF^+^ cells, 60 ± 2% PECAM-1^+^ cells, and 61 ± 1% VE-cadherin^+^ cells (Figure 4A,D). Increasing the concentration of Ang II to 25 or 50 ng/ml together with 2 ng/ml VEGF-A demonstrated no further significant increase in the percentage cells positive for the EC markers (Figure 4B,C,D).

### Differentiated MSCs form cobblestone morphology and capillary tubes

**Figure 5:**
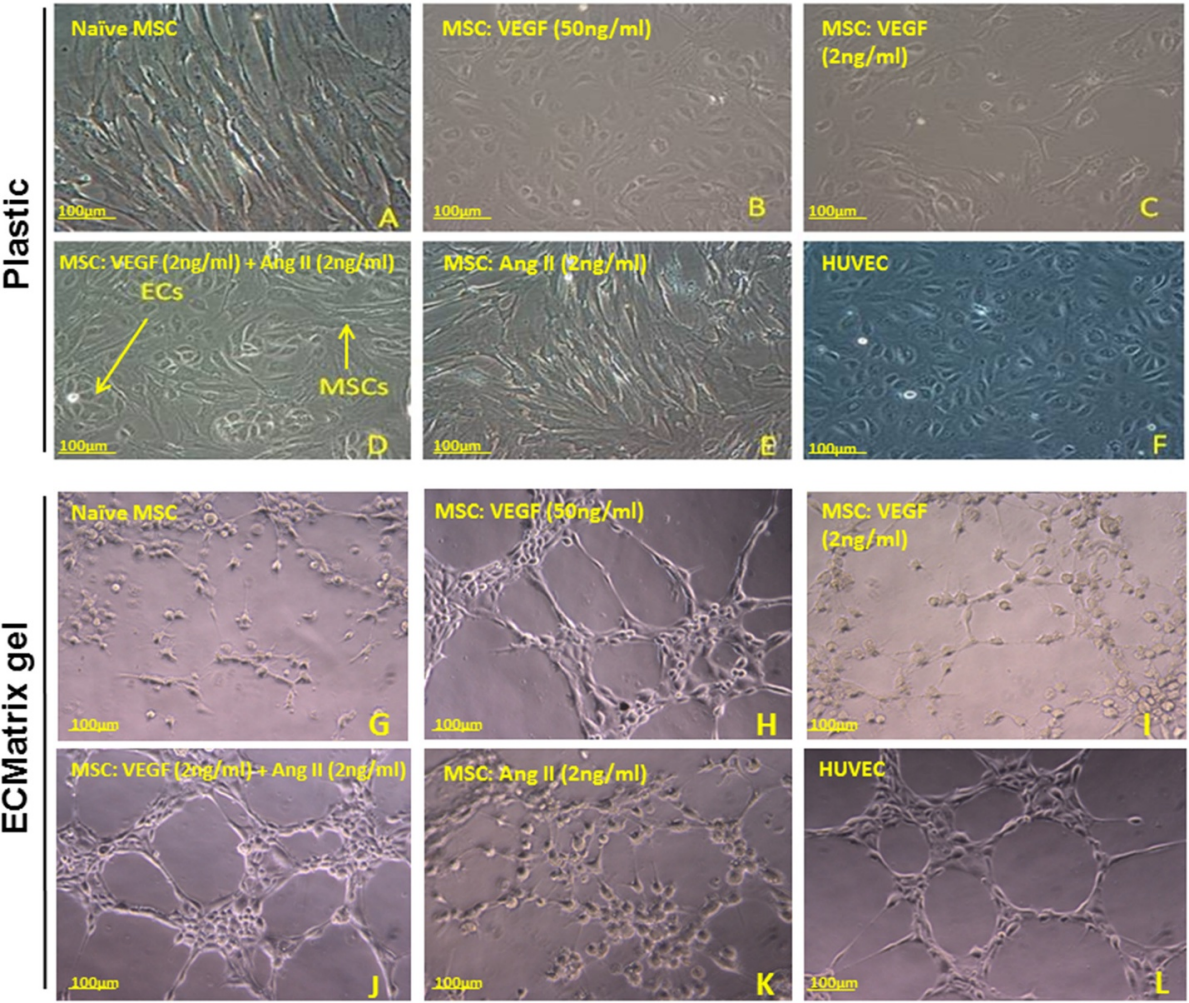
**Cell morphology of differentiated mesenchymal stem cells into endothelial cells.** Images taken by inverted microscope after 10 days of stimulation. Control mesenchymal stem cells (MSCs) cultured in growth media retained fibroblastoid morphology **(A)**. MSCs differentiated in high-dose vascular endothelial growth factor (VEGF-A) changed into the cobblestone shape typical of endothelial cells **(B)**; however, low-dose VEGF-A produced less of an effect **(C)**. Combination treatment with low-dose VEGF-A and low-dose angiotensin II (Ang II) also induced cobblestone morphology **(D)**, but Ang II alone had no effect **(E)**. Positive control (human umbilical vein endothelial cells (HUVECs)) demonstrated rigid cobblestone morphology **(F)**. Capillary formation by differentiated MSCs into endothelial cells (ECs) **(G, H, I, J, K, L)**. After 10 days of stimulation, cells were seeded onto ECMatrix gel. Naïve MSCs cultured in control media and incubated on the matrix did not form elliptical structure **(G)**. High-dose VEGF-A was capable of forming characteristic capillary structures associated with EC regeneration **(H)**. MSCs differentiated in low-dose VEGF-A started forming elliptical structures. However, individual cells did not connect with each other **(I)**. Combination treatment with low dose VEGF-A and Ang II resulted in complex mesh-like formation in addition to closed polygonal structure **(J)**. Ang II alone did not induce any mesh-like structure or connections **(K)**. The effect of the combined treatment was more striking compared with HUVEC cells forming capillaries **(L)**. One image representative of three independent experiments performed from three different swine bone marrow samples is shown.

Classically, naïve MSCs display a fibroblastic morphology [22], whereas ECs display cobblestone morphology [22]. Naïve MSCs cultured in EGM-2 control media retained a fibroblastoid shape (Figure 5A). High-dose VEGF-A (50 ng/ml) induced the cobblestone shapes (Figure 5B); however, low-dose VEGF-A (2 ng/ml) produced much less of an effect (Figure 5C). Combined treatment with low-dose VEGF-A (2 ng/ml) and Ang II (2 ng/ml) resulted in nests of cobblestone-shaped cells (Figure 5D). Ang II (2 ng/ml) alone had no effect (Figure 5E). HUVECs demonstrated rigid cobblestone interconnections (Figure 5F).

Next, we examined the higher order formation of capillary tube structures typical of endothelium maturation. After 10 days of stimulation, cells were seeded onto ECMatrix gel to assess angiogenesis. Naïve MSCs cultured in basic EGM-2 control media and incubated on ECMatrix gel did not form capillary structures (Figure 5G). MSCs differentiated in high-dose VEGF-A were capable of forming characteristic capillary structures (Figure 5H), similar to the positive control HUVECs (Figure 5L). In contrast, MSCs differentiated in low-dose VEGF-A (2 ng/ml) showed weak signs of capillary tube formation (Figure 5I). Combined treatment with low-dose VEGF-A (2 ng/ml) and Ang II (2 ng/ml) resulted in complex mesh-like formation in addition to closed polygonal structures (Figure 5J). Ang II (2 ng/ml) alone had no effect on capillary formation (Figure 5K).

### AT2R antagonist inhibits angiotensin II-stimulated endothelial cell differentiation

**Figure 6:**
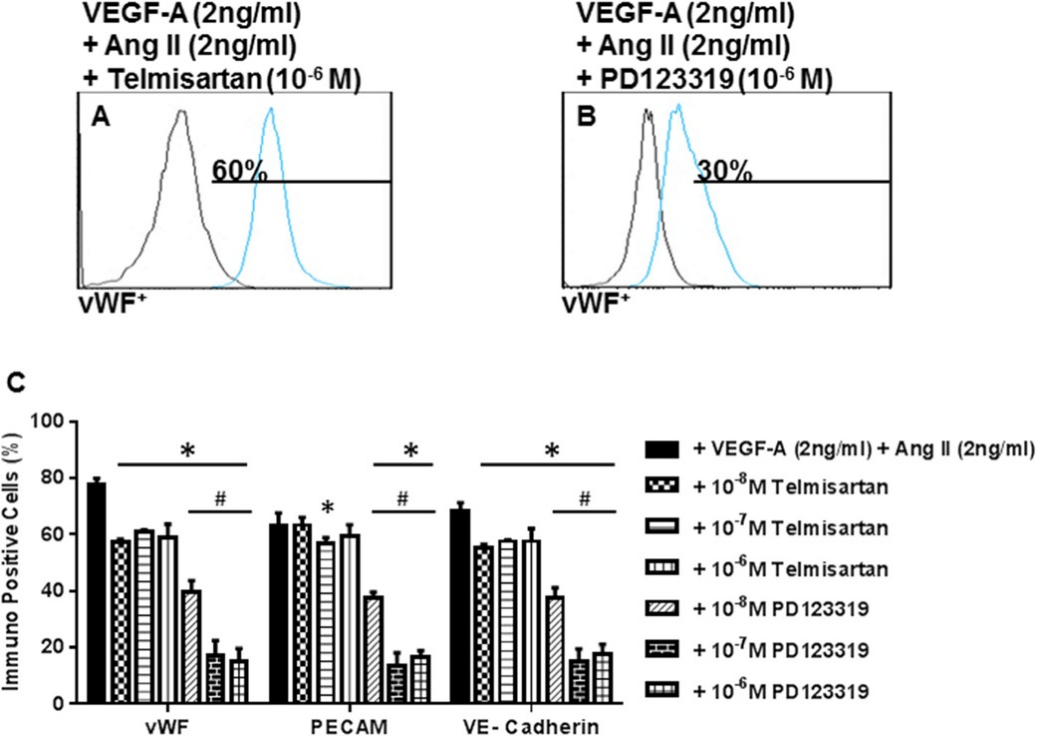
**Effect of antagonists for AT1R and AT2R on immunopositivity for endothelial cell markers.** Mesenchymal stem cells (MSCs) were pretreated with either angiotensin receptor AT1R or AT2R inhibitor and then cultured with angiotensin II (Ang II) and vascular endothelial growth factor (VEGF-A). PD123319, an AT2R inhibitor, significantly reduced the expression of platelet endothelial cell adhesion molecule-1 (PECAM-1), von Willebrand factor (vWF) and vascular endothelial cadherin (VE-cadherin) **(B)**, whereas there was a lesser effect of the AT1R inhibitor telmisartan **(A)**. Bar diagram showing data from six individual experiments **(C)**. Note that human umbilical vein endothelial cells were excluded from the statistical analyses. *P <0.05 vs. VEGF-A (2 ng/ml) plus Ang II (2 ng/ml)-treated MSCs. ^#^ *P* <0.05 telmisartan vs. PD123319-treated MSCs, *n* = 3.

Ang II is capable of transducing its effects via AT1R and AT2R. In order to determine whether AT1R or AT2R is pivotal to Ang II-mediated induction of EC marker expression, we utilized selective receptor antagonists. Naïve MSCs were pretreated with 5 μM of either inhibitor for 1 hour. Subsequently, the naïve MSCs were cultured in differentiation media containing VEGF-A (2 ng/ml) and Ang II (2 ng/ml) plus either 1 μM telmisartan or 1 μM PD123319 for 10 days. Our results showed that telmisartan (10^-6^ M) slightly diminished immunopositive expression of EC markers with 59 ± 2% vWF^+^ cells, 58 ± 1% PECAM-1^+^ cells, and 57 ± 6% VE-cadherin^+^ cells (Figures 6A,C). Conversely, PD123319 (10^–6^ M) significantly attenuated the induction of EC immunopositive phenotype by Ang II/VEGF-A to a level of 27 ± 1% vWF^+^ cells, 31 ± 3% PECAM-1^+^ cells, and 28 ± 2% VE-cadherin^+^ cells (Figure 6B,C). These data strongly support the critical role of AT2Rs in the synergistic effect of Ang II with VEGF-A in MSC differentiation into ECs.

### Angiotensin II-induced VEGF secretion does not account for MSC differentiation into endothelial cells

**Figure 7:**
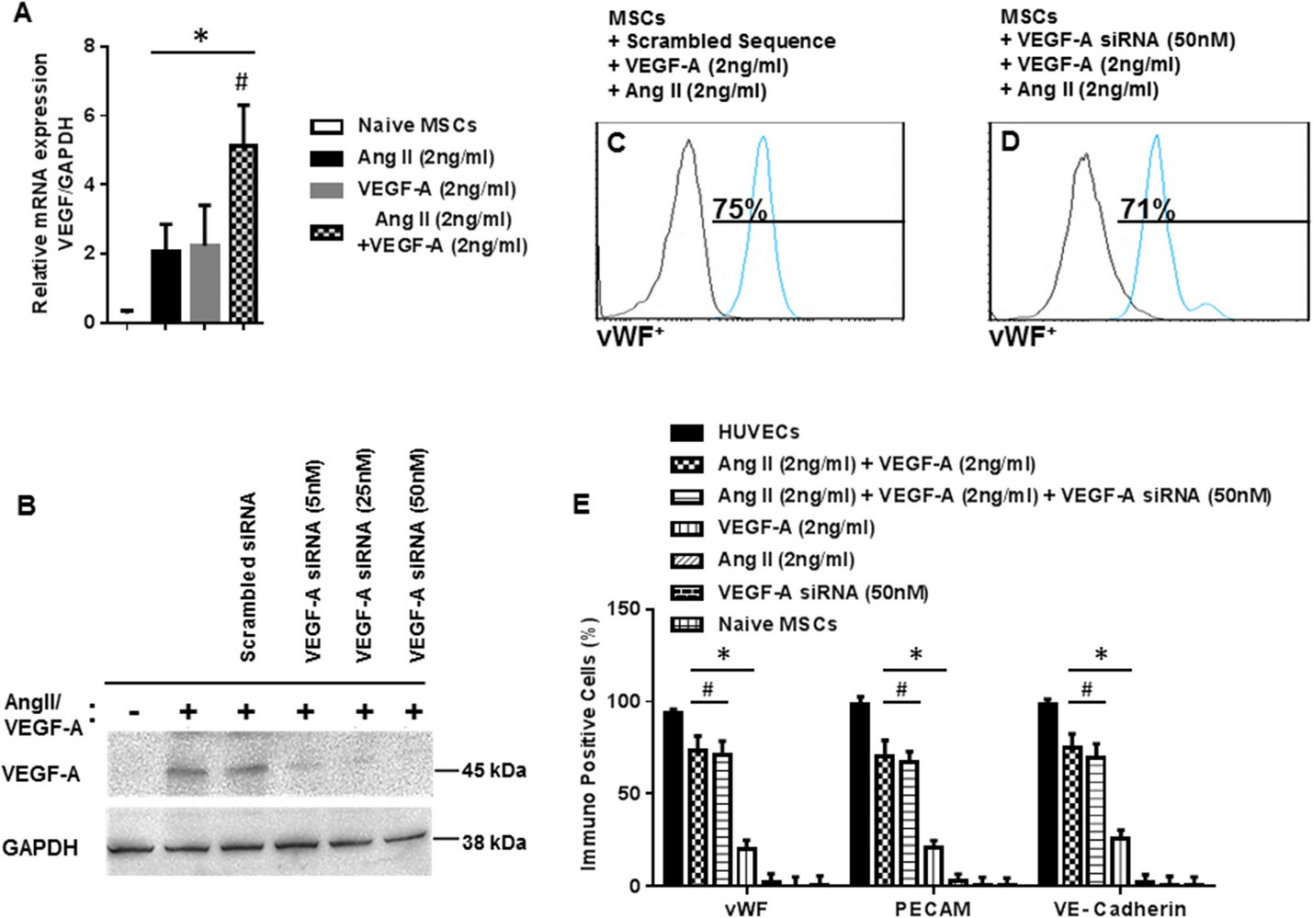
**Silencing of endogenous VEGF-A lacks an effect on mesenchymal stem cell differentiation into endothelial cells.** Differentiating mesenchymal stem cells (MSCs) were transfected with small interfering RNA (siRNA) directed against vascular endothelial growth factor (VEGF-A) and then examined for endothelial cell (EC) marker expression. Treatment of MSCs with the cocktail of VEGF-A (2 ng/ml) plus angiotensin II (Ang II; 2 ng/ml) for 24 hours induced a synergistic increase in VEGF transcript as compared with VEGF-A alone or Ang II alone **(A)**. VEGF-A mRNA was normalized to glyceraldehyde-3-phosphate dehydrogenase (GAPDH) mRNA (A). Standardization experiments demonstrated that 50 nM siRNA directed against VEGF completely abrogated VEGF protein expression **(B)**. Fluorescence-activated cell sorting analysis revealed that there was no significant difference in EC marker expression between differentiating MSCs that received VEGF-A siRNA or scrambled control siRNA **(C, D, E)**. Note that human umbilical vein endothelial cells (HUVECs) were excluded from the statistical analyses. *P <0.05 vs. naïve MSCs. ^#^ *P* <0.05 vs. VEGF-A (2 ng/ml)-treated MSCs or Ang II (2 ng/ml)-treated MSCs alone, *n* = 3.

Previous studies have demonstrated that Ang II stimulates the synthesis of VEGF in rat BM-MSCs through an AT1R-dependent mechanism. Thus, we tested VEGF-A mRNA expression in MSC cultures stimulated with Ang II (2 ng/ml) alone, VEGF-A (2 ng/ml) alone, or the combination of VEGF-A/Ang II for 24 hours. Treatment with either Ang II or VEGF-A significantly upregulated VEGF-A mRNA compared with naïve MSCs (Figure 7A). Furthermore, the combination of VEGF-A and Ang II produced an even greater induction VEGF-A mRNA (Figure 7A).

To address the role of endogenous VEGF-A in MSC differentiation, experiments using siRNA directed to VEGF-A were performed. siRNA-mediated knockdown of endogenous VEGF-A was confirmed by western blot of protein lysates from MSCs stimulated with VEGF-A (2 ng/ml) plus Ang II (2 ng/ml) (Figure 7B). Knockdown of endogenous VEGF-A failed to prevent induction of EC marker expression during differentiation with the combination treatment of exogenous VEGF-A plus Ang II (Figure 7C,D,E).

## Discussion

MSCs offer remarkable potential in regenerative medicine and are currently being investigated for the treatment of cardiovascular disease [27, 28]. MSCs are present in various organs, including the bone marrow, adipose tissue, liver, and spleen [25]. MSCs are characterized as a plastic adherent population, expressing CD44, CD73, CD90, and CD105 and lacking CD11b, CD19, CD34, or HLA-DR surface molecules [29–31]. MSCs can be differentiated into cells of the mesoderm, including osteocytes, chondrocytes, and adipocytes [29, 30]. Strategies for differentiating BM-MSCs into ECs have therapeutic potential in cardiovascular diseases and tissue engineering [30, 31].

Our group and others have shown that VEGF-A is capable of stimulating the differentiation of MSCs or progenitor ECs into ECs [22, 32]. However, the mitogenic effects of VEGF-A make it unsuitable for therapeutic applications, especially at high doses. The novel finding of this study is that AT2R mediates the synergistic effect that the combination of Ang II and VEGF has to promote differentiation of swine BM-MSCs into ECs. Our data suggest that AT2R could be critical to repair and/or regeneration of damaged vascular endothelium.

Intriguingly, it has been observed that Ang II levels are increased during occlusive vascular pathologies where the endothelium is severely injured and denuded, such as in balloon angioplasty [33]. At the site of injury, MSC traffic increases and AT1R and AT2R expression can become upregulated [31]. An increase in the local production of Ang II acts to stimulate proliferation of vascular smooth muscle cells and constriction of vascular muscle via activation of AT1R. In terms of AT2R, results from this study suggest that Ang II may also act on AT2R to stimulate repair or regeneration of the damaged endothelium.

A study by Shi and colleagues investigated the effects of Ang II on rat BM-MSCs [33]. The study found that treatment with Ang II (100 nM; equivalent to ~105 ng/ml) induced VEGF production in MSC culture media from approximately 140 pg/ml to 350 pg/ml [33]. According to our data using a low-dose of VEGF-A (2 ng/ml), such a low level alone is not effective in upregulating EC marker expression or in promoting MSC differentiation into ECs. Furthermore, we found that siRNA-mediated knockdown of endogenous VEGF-A did not alter the induction of EC marker expression mediated by the combination of exogenous VEGF-A plus Ang II. Thus, although Ang II and VEGF-A stimulate the endogenous production of VEGF, this effect has only a minor contribution to MSC differentiation into ECs. As a case in point, treatment of MSCs with Ang II alone was not sufficient to induce EC marker expression. It is also important to consider that it is necessary under the differentiation protocol for the media to be changed every 48 hours in order to maintain cell viability. The effects of endogenous production of VEGF and other factors that accumulate in the conditioned media are nullified each time the cells are switched to fresh media.

Shi and colleagues also demonstrated that Ang II-stimulated VEGF production by rat BM-MSCs was inhibited much more by the AT1R antagonist losartan than the AT2R antagonist PD123319 [33]. Mechanistically, Ang II-mediated induction of VEGF was shown to occur through extracellular signal regulated kinase (p42/44, ERK1/2) and Akt pathways via AT1R. Results from the current study indicate that AT1R inhibition caused only a slight reduction in EC marker expression upon co-treatment with VEGF-A/Ang II. Rather, AT2R plays a major role since treatment of MSCs with the AT2R antagonist PD123319 (10^–6^ M) caused a marked attenuation of EC marker induction by differentiation in the presence of exogenous Ang II/VEGF-A. The specific signal transduction pathways downstream of AT2R activation with regard to the MSC versus EC phenotype are not currently well understood.

ATR-mediated and VEGF receptor-mediated signaling involves downstream mediators including c-Jun NH_2_-terminal kinase, p38 mitogen-activated protein kinase, [34] ERK1/2 [35], and SHP-1 [35]. In particular, AT2R-Gαi stimulates the production of prostaglandins and nitric oxide [36, 37]. Interestingly, nitric oxide is reported to augment EC differentiation [38]. These candidate pathways conceivably contribute to the mechanisms by which the combination of Ang II and VEGF-A drives MSC differentiation into ECs. By immunofluorescence, we observed that only a fraction of naïve MSCs express AT1R and AT2R. This suggests that AT2R-dependent induction of EC differentiation may be associated with secretion of mediators from AT2R-expressing cells. Ultimately, such mediators could have paracrine effects that lead to large-scale differentiation of MSCs into ECs.

Further investigation is needed to determine whether or not EC-like cells derived and differentiated from MSCs are functional in all aspects of EC biology, including leukocyte/platelet adhesion and the production of vasoactive substances. With regard to angiogenesis, studies have shown that both Ang II and VEGF-A promote capillary tube formation, a distinctly EC trait [37, 38]. Likewise, a pro-angiogenic role for AT2R signaling has been demonstrated using AT2R-KO mice or mice treated with AT2R antagonist. Results from the current study also support a pro-angiogenic role for AT2R [39].

## Conclusions

**Figure 8:**
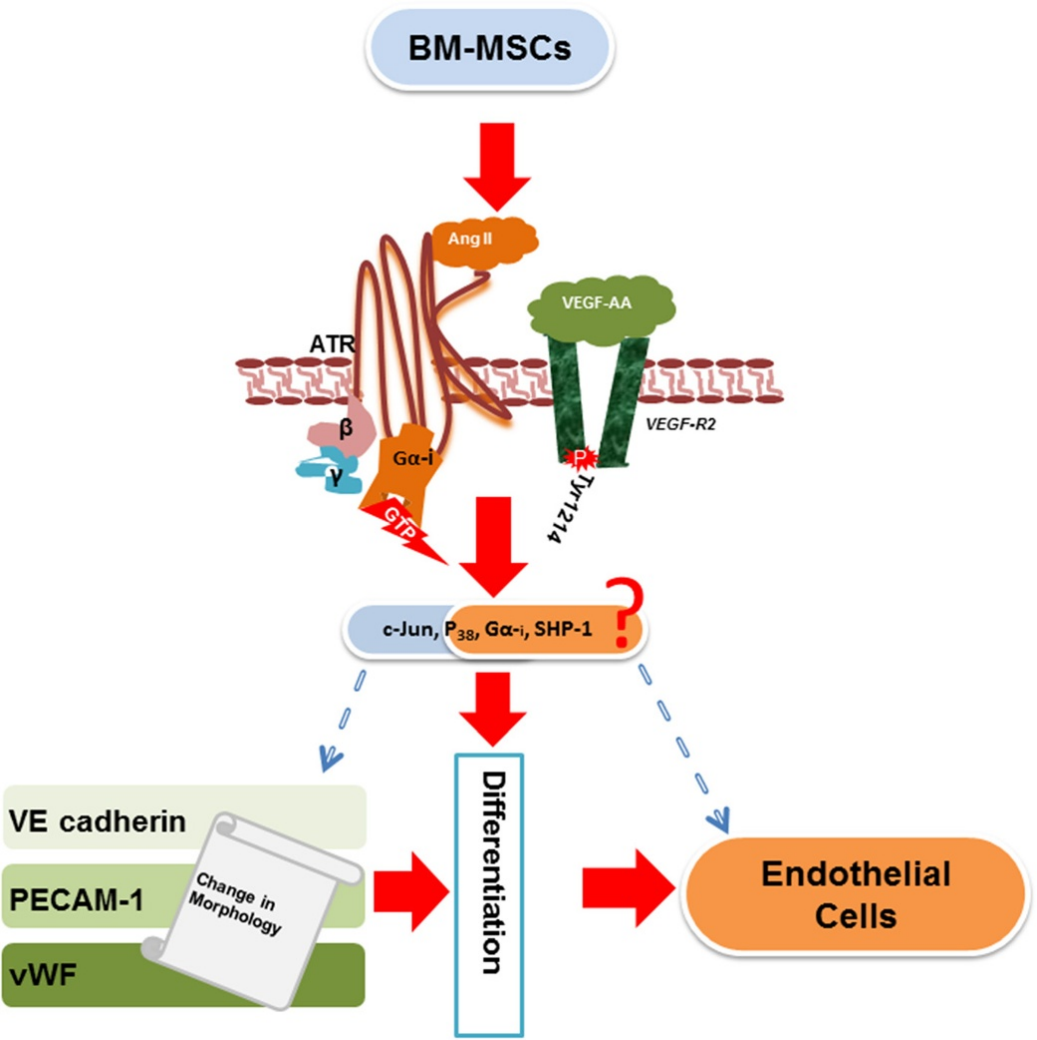
**Summary of synergistic effect of angiotensin II on VEGF-A-mediated differentiation of BM-MSCs into endothelial cells.** Ang II, angiotensin II; ATR, angiotensin receptor; BM, MSC, bone-marrow-derived mesenchymal stem cells; PECAM-1, platelet endothelial cell adhesion molecule-1; VE-cadherin, vascular endothelial cadherin; VEGF, vascular endothelial growth factor; vWF, von Willebrand factor.

In summary (Figure 8), Ang II-dependent signaling mediated by AT2R plays a critical role in the differentiation of BM-MSCs into ECs. Results from this study highlight the importance of investigating AT1R-specific effects versus AT2R-specific effects on EC function and differentiation. The knowledge gained from this study is of clinical importance, given the prevalence of the use of angiotensin-converting enzyme inhibitors and angiotensin receptor blockers in the treatment of patients with vascular disease. Connecting EC differentiation to AT2R signaling could have immediate clinical and translational application.

Drug-based therapies for cardiovascular diseases that impair AT2R function may have an unintended effect on vascular re-endothelialization. For instance, drugs that prevent formation of Ang II, including angiotensin-converting enzyme inhibitors [40, 41], could have negative consequences on MSC differentiation into ECs, since Ang II is an endogenous agonist of AT2R. It is feasible that AT2R-specific agonists could be beneficial as a supplementary treatment in these cases. Moreover, a better understanding of AT2R and VEGF-A mechanisms in EC differentiation could be instrumental in the development of cell-based therapies towards the re-endothelialization necessitated by interventional procedures.

## Abbreviations

Ang II: angiotensin II
ATR: angiotensin receptor
BM-MSC: bone marrow-derived mesenchymal stem cell
DM: differentiating media
EC: endothelial cell
EGM-2: endothelial growth media 2
GAPFH: glyceraldehyde-3-phosphate dehydrogenase
HUVEC: human umbilical vein endothelial cell
MSC: mesenchymal stem cell
PBS: phosphate-buffered saline
PECAM-1: platelet endothelial cell adhesion molecule-1
siRNA: small interfering RNA
VE-cadherin: vascular endothelial cadherin
VEGF: vascular endothelial growth factor
vWF: von Willebrand factor.
